# The Effect of the Lunar Cycle on Fecal Cortisol Metabolite Levels and Foraging Ecology of Nocturnally and Diurnally Active Spiny Mice

**DOI:** 10.1371/journal.pone.0023446

**Published:** 2011-08-04

**Authors:** Roee Gutman, Tamar Dayan, Ofir Levy, Iris Schubert, Noga Kronfeld-Schor

**Affiliations:** Department of Zoology, Tel Aviv University, Tel Aviv, Israel; Vanderbilt University, United States of America

## Abstract

We studied stress hormones and foraging of nocturnal *Acomys cahirinus* and diurnal *A. russatus* in field populations as well as in two field enclosures populated by both species and two field enclosures with individuals of *A. russatus* alone. When alone, *A. russatus* individuals become also nocturnally active. We asked whether nocturnally active *A. russatus* will respond to moon phase and whether this response will be obtained also in diurnally active individuals. We studied giving-up densities (GUDs) in artificial foraging patches and fecal cortisol metabolite levels. Both species exhibited elevated fecal cortisol metabolite levels and foraged to higher GUDs in full moon nights; thus *A. russatus* retains physiological response and behavioral patterns that correlate with full moon conditions, as can be expected in nocturnal rodents, in spite of its diurnal activity. The endocrinological and behavioral response of this diurnal species to moon phase reflects its evolutionary heritage.

## Introduction

Rodents are vulnerable to predation [1]–[3]. As most rodent species are nocturnal [4], owls, many of which are small mammal specialists, pose a serious threat. While earlier ecological studies have focused on the population dynamics of predator-prey relationships, the past two decades have seen a growing realization that behavioral responses of prey to predation risk have further profound ecological implications (e.g., [5]–[7]). Therefore, considerable research has been devoted to recording rodent behavioral responses to direct and indirect cues of owl predation risk and to understanding their ecological implications (e.g., [8]–[14]). Among indirect cues, particular attention has been aimed at the response of rodents to moon phase as a surrogate for predation pressure (e.g., [9], [11], [15]–[20]).

Owl strike success rate is high [21], particularly under elevated illumination [14], [22], and successful strikes are invariably lethal. As learning from experience is simply too costly, accurate perception of predation risk from owls and relevant behavioral responses may be predominantly genetically determined. Indeed, captive-bred individuals that have never encountered owls in nature were repeatedly found to respond behaviorally to changes in predation risk and illumination levels (e.g., [23]–[26]). The mechanism underlying these responses may include the elevation of glucocorticoids (GC). In this case, nocturnal rodents will respond adaptively to elevated illumination during full moon nights, by increasing GC levels that cause a reduction in foraging and therefore in predation risk. It has been suggested that apart from being a response to the stressor itself, GCs have a role in preparing the individual to an expected stressor [27]. In these cases, GC concentrations increase in anticipation of a challenge, rather than in response to the challenge itself. Such preparative action requires that the stressor (predation in the current work) will be statistically predictable [27]–[29]; because moonlight increases predation risk, and because elevated moonlight occurs with regularity in the lunar cycle, risk of predation by nocturnal raptors is entirely predictable, and it would be attractive to hypothesize that a lunar rhythm in stress hormones has evolved in response (but see Discussion).

In laboratory biomedical experiments using rodents, light is routinely used as a cue for stress. Light pulses during the night were shown to cause an increase in GC levels in rats [30], GC treatment was shown to increase anxiety and conditioned fear [31]–[34], and acute corticosterone elevation enhanced antipredator behaviors in tree lizard species [35]. At least some of these responses may be genetically determined; experimental studies in laboratory rodents as well as in captive-bred wild species that have never encountered predators have shown that exposure to light, a predator, or even its voice or odors, can cause an increase in stress hormone levels (e.g., [30], [35]–[38]). In some cases increased GC levels led to increases in food intake, in particular of highly palatable foods [39], [40], but in most cases increased GC levels result in a decrease in food intake (reviewed by [41]). The effect of lunar phase on rodent activity under natural conditions at night has been studied extensively by ecologists, the role of light as a stressor is clear on experimental grounds, and the connection between the behavioral response to risk and stress hormones is well established; however the effect of lunar phase on stress hormones in rodents in the field was never studied.

Although most current mammals retain the mammalian ancestral state of nocturnality, species within both closely and distantly related taxa have evolved to become diurnal (reviewed by [42]). This shift released many species from the threat of owl predation, exposing them to diurnal predators, whose predation efficiency is not influenced by lunar cycle. However, since predation by owls is a strong selective force which has produced ‘hard-wired’ endocrinological and behavioral responses, diurnally active rodents may retain these responses. Alternatively, these adaptations may be lost with release from natural selection (e.g., [43], [44]).

We studied lunar cycle patterns of stress hormone levels and foraging behavior of nocturnal and diurnally active rodents asking whether stress hormone levels correlate with moon phase, whether foraging behavior correlates with moon phase, and whether they do so both during the day and during the night. *Acomys russatus* is a diurnally active rocky desert rodent but in absence of its nocturnal congener (*A. cahirinus*) it is also active at night [45]–[47]. Molecular phylogenetic research suggested that the *A. russatus* lineage diverged ca. 6–8 million years ago, but it has been suggested that the shift to diurnality occurred at the evolutionary scale ca. 0.3–0.5 million years ago when it encountered the younger lineage *of A. cahirinus* [48, and Volobouev pers. com.].

In the field, spiny mice are preyed upon at night by Blanford's foxes (*Vulpes cana*, although they form only a small part of the fox's diet, [49]), owls (Hume's Tawny Owl, *Strix butleri* [50]), and snakes (saw-scaled viper, *Echis coloratus*); during the day they are probably preyed upon by diurnal raptors (Common Kestrel, *Falco tinnunculus*) [51]. General indirect evidence for the evolutionary significance of predation are spines on spiny mouse rumps, in particular those of *A. russatus*, a histological mechanism for tail loss [52], and relative immunity to viper venoms [53].

*A. cahirinus* reduce their foraging activity as a result of predation risk by owls in open habitats during moonlit nights, as has been demonstrated for rodents of sandy deserts [19]. In response to owl calls, the level of stress hormones of *A. cahirinus* increases [23], and their motor behavior changes with rising illumination levels.

Nocturnal Blanford's foxes pose a risk of predation in open areas, regardless of moon phase, probably reinforcing preference of sheltered microhabitats driven primarily by owl predation [51], [54].

The saw-scaled viper is active at Ein Gedi during the warm summer months. Predation by vipers is a threat primarily under boulders during the day (where these nocturnal sit-and-wait predators rest curled up); during night, it is a threats both under and between boulders and in open areas, habitats where snakes either lie still or move actively at night [55]. Consequently both spiny mouse species reduce their foraging in sheltered microhabitats, and shift their foraging activity to more open microhabitats in summer [51]. Thus, in summer the selective pressure posed by vipers counters that of owl predation risk during night (see also [56] and that of physiological stress during the day, regardless of moon phase.

In sum, being active during the day poses different challenges to *A. russatus* than being active at night, among them differences in predation regimes [19], [51], [54]. Specifically, risk of predation by owls should be a threat only during the night. Indeed, analysis of pellets of the resident owl at Ein Gedi, the tawny owl, confirms that in nature owls take only nocturnal *A. cahirinus* [19]. In response, *A. cahirinus* prefer to forage in sheltered microhabitats where they are safe from avian predators [51] and reduce their foraging in the open during moonlit nights [19].

Light increases activity in diurnal mammals (positive masking) and suppresses it in nocturnal ones (negative masking), while darkness acts in the opposite way [57]–[60]. In order for a nocturnal species to evolve into a diurnal one or for an individual to move from a nocturnal to a diurnal activity niche, the negative masking effects of light on activity must be overcome. In accord, masking effect of light in rodent species which show individual differences in activity patterns, was associated in predictable ways with the overall activity pattern that the individuals exhibited at the time of testing [57]–[60]. In previous experiments we found that light suppresses activity and body temperature levels of *A. cahirinus* in the laboratory as well as in the field (negative masking), but not in *A. russatus* [61], [62].

It appears that *A. russatus* is a nocturnal species forced into diurnality; it retains several nocturnal physiological and morphological traits, including nocturnal circadian rhythms, but it has evolved also some morphological and physiological adaptations for diurnal activity [42], [46], [61], [63]–[69]. Is its response to lunar phase also evolutionarily constrained? Will *A. russatus*, active in nature only during the day [46], [67], respond to the lunar cycle when a manipulative field experiment enables it to extend activity into the night? If so, will stress hormone levels and diurnal activity patterns respond to moon phase as well?

In order to address these questions, we carried out a field study, including a controlled and replicated removal experiment in field enclosures, enabling us to gain insight into the role of the lunar cycle on stress hormones levels and on foraging behavior (see also [45]) during day and night. Specifically, we compared fecal stress hormone levels and nocturnal foraging behavior of *A. cahirinus* and *A. russatus* during new moon and full moon nights and diurnal foraging behavior of *A. russatus* in the days that follow them.

We framed our working hypotheses as follows:

1. *A. cahirinus* stress hormone levels will increase, and it will reduce foraging behavior on full moon nights.
2. If *A. russatus* retains its response to moon phase, then when a manipulative field experiment enables it to extend its activity into the night, it will respond to the lunar cycle in a similar manner to *A. cahirinus*; its stress hormone levels will increase, and it will reduce foraging behavior on full moon nights.
3. If that is the case, *A. russatus* stress hormone levels and diurnal foraging behavior may be affected by moon phase as well, in either an adaptive way (decreased stress hormone levels and increased diurnal foraging to compensate for reduced nocturnal foraging) or a maladaptive way that reflects an evolutionary constraint (increased stress hormone levels and decreased foraging during the day in response to moon phase).
4. Our alternative hypothesis was that the endocrinological and behavioral response to moon phase has been lost in this diurnally active species and that *A. russatus* behavior and stress hormones levels will not be affected by moon phase.

## Materials and Methods

The study was conducted in four research enclosures, 2 populated with both *A. cahirinus* and *A. russatus*, and two populated with *A. russatus* only (for details see below), as well as in free ranging populations of both species (for hormone levels only).

### Populating experimental enclosures

We conducted the study at the Ein Gedi Nature Reserve, in the Judean Desert near the Dead Sea (31°28′N, 35°23′E, 300 m below sea level). We erected four mouse-proof enclosures (20×50 m) over linear rock terraces (see details and feeding regime in [45]). As we could not be certain that the food and water naturally available in the enclosures were sufficient to sustain the spiny mice, a limited amount of food (commercial rodent pellets and sunflower seeds) was added every two weeks at the end of each trapping session, and between foraging experiments (see protocol bellow). Food was not supplemented at the time of the experiments. Rodents in the enclosures are exposed to the same suite of predators they face in nature in this area (foxes, snakes, owls, and probably raptors) [52]–[54].

We captured spiny mice at the Ein Gedi area in fall (1999), marked them individually, and introduced them into the enclosures (from which resident individuals were trapped and removed), allowing them several months to acclimate. The first experiment took place during June and July, 2000. We placed Sherman live traps in the enclosures for at least two consecutive days and nights every other week; individuals of both species can be easily and repeatedly trapped and both show similar trappability [70]. Traps were closed before sunrise and sunset, checked for trapped rodents, and then reopened only after sunrise or complete darkness. By doing this, we could monitor population size and clearly define the time of activity (nocturnal or diurnal).

We removed or released surplus mice that were born in the enclosures to regulate population size, held constant as from *ca.* 6 months prior to the experiment. We monitored the sex ratios, kept at *ca.* 1∶1 for each species. Foraging experiments began in June (2000) after trapping results indicated a shift in activity patterns of *A. russatus* held alone (see [45]); there were 8 individuals of each species in the two species enclosures and 16 individuals of *A. russatus* in the *A. russatus* enclosures.

### Fecal stress hormones

#### Fecal cortisol metabolite levels of free living spiny mice

We trapped free-living spiny mice using 100–200 Sherman live traps during full and new moon nights during December–June 2003–2004 (*A. russatus*: new moon n = 19, full moon n = 12; *A. cahirinus*: new moon n = 18, full moon n = 22). Traps were set at different areas in each sampling session, and mice were marked with paint before they were released in order to avoid re-sampling. Only one fecal sample was collected from each individual. Sampling sessions lasted 24–72 h, dependent on trapping success. *A. russatus* were always trapped during the day, while *A. cahirinus* were always trapped during the night. Trapping is a stressor; in spiny mice, trapping stress is evident in the fecal stress hormone metabolite levels after 8–12 h (see Text S1). Traps that were opened at sunset and sunrise were checked 6–8 or 4–6 h later, respectively, to avoid measuring trapping stress. Traps opened at sunrise were checked 4–6 hours later to avoid heat stress to the animals. Hence, fecal stress hormone levels reflect the time interval before the animals entered the traps. Feces were collected from the traps and stored in 95% ethanol in −20°C until extraction.

#### Stress hormone levels in the enclosures

During June and July 2003, we trapped individuals in the *A. russatus* enclosures during days following full moon (n = 12) and new moon (n = 12) nights, using 10 Sherman live traps in each enclosure. Each individual was sampled once during each moon phase. Traps were opened at sunrise and checked 4–6 h later. Food was not supplemented at the time of the feces collection.

#### Hormone extraction

Hormones were extracted based on Harper and Austad [71]. Before extraction, feces were dried in open air to constant weight. 10 ml of ethanol were added to each sample, and the samples were boiled for 20 minutes at 90°C. The ethanol was transferred to a new tube, another 10 ml of ethanol were added to the original tube, and boiled again. The ethanol from the two boiling sessions was combined and dried using a sample concentrator (Techne) using nitrogen at 60°C (heat block, Ori-block 08-3), rinsed with ethyl acetate: hexane (3∶2, v/v) solution, and dried again. Samples were stored in −20°C until analyzed.

In spiny mice, unlike most rodents, the major glucocorticoid is cortisol [23], [72]. Fecal cortisol metabolite was measured using a tritiated RIA kit for unextracted serum (ICN biomedical, inc., catalog number 07-221105) in duplicates. The assay was validated for both species: Biological validity, which included two experiments; 1. Serial sampling before and after a stressful event (trapping, including a control group without stress), which showed that trapping stress causes a significant effect on fecal cortisol metabolite levels after 8–12 h, demonstrating that the technique can detect biologically meaningful changes in GC levels (paired t-tests: *A. russatus*: 4 hr: t = −0.33, df = 14, p = 0.37; 8 hr: t = −1.15, df = 14, p = 0.13; 12 hr: t = −2.61, df = 11, p<0.05, *A. cahirinus*: 4 hr: t = −0.81, df = 14, p = 0.21; 8 hr: t = −0.33, df = 13, p = 0.51; 12 hr: t = −1.49, df = 13, p = 0.07; Figure S1). 2. Describing the naturally occurring diurnal variation in the fecal cortisol metabolite levels, which also indicate biological relevance. Protocol validity: cortisol (from standard solutions) and cortisol-like immuno-reactivity in spiny mice feces extract diluted in parallel in the ICN cortisol RIA (p = 0.0052, R^2^ = 0.817 for *A. russatus* and p = 0.0083, R^2^ = 0.828 for *A. cahirinus*; Figure S2). We also extracted an increasing amount of homogenized pool of fecal matter and found a correlation between fecal mass extracted and fecal cortisol metabolite levels in the extract (simple regression analysis: *A. russatus* mass effect −1430.9±291.1, R^2^ = 0.83, t = 4.9, df = 5, p<0.01; *A. cahirinus* mass effect −698.3±127.1, R^2^ = 0.86, t = 5.5, df = 5, p<0.01; [µg/dL±SD]; Figure S3. For all validation methods and results Text S1). Our within-assay coefficients of variation were 0%–5%, and the between assay coefficient of variation was 6.5%. Before analysis, samples were re-constituted using 100–500 µl ethanol, according to their fecal mass. The dilution factor was taken into account in the concentration calculation. We used a control solution (BIO-RAD liphocheck immunoassay plus control) as a quality control.

### Studying foraging behavior with artificial food patches

We studied foraging behavior of spiny mice using the giving-up density method (GUD, [73]), which assumes that a forager is behaving optimally and that the density of food remaining in the patch when it gives up foraging corresponds to a harvest rate at which the energetic gain from foraging just balances the metabolic cost of foraging, the cost of the perceived risk of predation in foraging in that patch, cost of interference, and the missed opportunity cost of not foraging elsewhere or indulging in other fitness enhancing activities [9], [17]. During both day and night, standard artificial food patches maintained similar metabolic costs of foraging associated with digging for food in the trays and similar missed opportunity costs from not foraging in other artificial food patches; therefore, differences between different days during the lunar cycle can be ascribed to differences in the perceived risk of predation. Predation risks during day and during night appeared constant across enclosures. Rarely, a viper settled for a day or two in one of the enclosures and dramatically affected foraging activity in the entire enclosure but not other enclosures. Such a case happened in the second new moon experiment. Therefore, we omitted the number of trays foraged in these days from our analysis.

Our artificial food patches comprised aluminum trays (30×20×4 cm) containing two liters of finely sifted local soil and two grams of crushed and sieved sunflower seed (1–2 mm length), mixed thoroughly and protected from foraging birds by heavy wire frames and fine filament fish netting (see [51] for details). Each experiment was preceded by three days and nights of pre-baiting to ensure that the trays had been discovered by rodents.

We placed nine trays in each enclosure, divided into 3 stations, 3 trays in each. The tray stations were placed *ca.* 12 m apart and trays in each station were placed in a triangle *ca.* 3 m apart from one another. We collected two types of data: a) number of trays foraged, as a measure of total activity level (since spiny mouse numbers were held constant across enclosures); b) giving-up densities, which should be independent of the number of mice that have visited the tray [73].

### Experimental protocol

We studied nocturnal foraging microhabitat use and efficiencies of *A. russatus* in absence of *A. cahirinus*, and of *A. cahirinus* on full moon and new moon nights (June and July). We also studied diurnal foraging of *A. russatus* during days following full and new moon nights. Four runs were conducted: two on full moon days and nights and two on new moon days and nights. Each experiment lasted a week: three days of pre-baiting and population monitoring using traps followed by four days of actual foraging experiments, when no trapping took place. We examined trays for footprints and GUDs measured at sunrise and at sunset, as in [51]. We determined identities of foragers in the two species enclosures (*A. russatus* or *A. cahirinus*) based on trapping results (discussed also in [45]). In brief, trapping results revealed that presence of *A. cahirinus* had a significant effect on activity times of *A. russatus* [45]. When kept together, *A. cahirinus* and *A. russatus* were temporally partitioned: *A. cahirinus* was nocturnal while *A. russatus* was diurnal [45]. In the *A. russatus* enclosures, *A. russatus* were trapped also during the night but still significantly more frequently during the day [45].

*A.russatus* night trappings showed that 10 different individuals were trapped during the night (of 13 night trappings), suggesting that nighttime activity was widespread among individuals of *A. russatus* in the *A. russatus* enclosures [45].

### Statistical analyses

We collected data from several trays at the same enclosures multiple times. As a result, if the same individual foraged in several trays on multiple occasions, these differences in GUD would not represent independent data points. We dealt with this issue by analyzing the trays data with the enclosures as the experimental units. We used mixed-effects modeling to analyze data because of the repeated measurements and the hierarchical nature of the sampling (trays sub-samples within each enclosure). Such models allow for the use of all data while correcting for pseudoreplication below the enclosure level (e.g. [74]). We used Bayesian inference because of the observational nature of the study [75] and ran the statistical models using a Markov Chain Monte Carlo (MCMC) simulation implemented in the JAGS computer program [76]. We used non-informative priors for all model parameters and used the R CODA software package [77] to calculate parameters' estimation (with standard deviations and 95%, 99%, and 99.5% confidence intervals [CI]), and to test their convergence (by convergence criteria described in detail in [78] and in [79]).

We analyzed (1) presence and absence of foraging on trays using generalized linear mixed-effects models (GLMM) (binomial data), (2) the GUD data using linear mixed-effects models (LMM), and (3) the amount of seeds eaten at each enclosure using LMM. In each model, we separated the statistical inference for each species: for *A. cahirinus*, we examined the effect lunar phase, and for *A. russatus* we examined the effect of competition (presence or absence of *A. cahirinus*), lunar phase, and activity phase. We then statistically compared the effect of moon phase between the species. At models 1 and 2, we added the enclosure type, enclosure index, line of trays, microhabitat, the day of measurement and the Julian month as random factors. At model 3, we added the enclosure type, enclosure index, the day of measurement and the Julian month as random factors.

We used deviance information criterion (DIC; [80]), which can be seen as the AIC Bayesian counterpart for model selection, to find whether each of the random factors contributed to the fit of the models. We found that all of the above random factors contributed to the models adequacy. We report estimates ± SD and the most significant level of CI. Two-way ANOVA on log transformed data was used for testing the effect of moon phase on fecal cortisol metabolite levels. The residuals of the LMMs and Two-way Anova models were normally distributed, based on graphical examination of their histogram (see Zuur et al. 2009).

## Results

**Figure 1:**
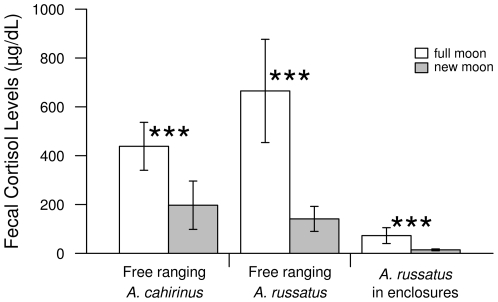
Fecal cortisol metabolite levels of free ranging diurnal *A. russatus* and nocturnal *A. cahirinus*, and of diurnal *A. russatus* in *A. russatus* enclosures during full moon and new moon nights and the following days (free ranging diurnal *A. russatus* new moon n = 19, full moon n = 12; free ranging nocturnal *A. cahirinus* new moon n = 18, full moon n = 22; *A. russatus* enclosures diurnal *A. russatus* new moon n = 9, full moon n = 8. *** p<0.001). Moon phase influenced fecal cortisol metabolite levels in both free ranging diurnal *A. russatus* and nocturnal *A. cahirinus*. In both, fecal cortisol metabolite levels were significantly higher in full moon nights than during new moon nights. A similar pattern was found in *A. russatus* in the *A. russatus* enclosures, where they are active both during the day and during the nights.

We found that moon phase influenced fecal cortisol metabolite levels in both free ranging diurnal *A. russatus* and nocturnal *A. cahirinus*. In both species, fecal cortisol metabolite levels were significantly higher in full moon nights and the days following them than during new moon nights and the days following them (df = 1, F = 16.7, p<0.001, Figure 1). A similar pattern was found in *A. russatus* in the two species enclosures, where they are active both during the day and during the night (no moon*enclosure/free interaction, df = 2, F = 2.4, p = 0.1, Figure 1). Fecal cortisol metabolite levels were significantly higher in free ranging spiny mice then in spiny mice in the enclosures (df = 2, F = 16.3, p<0.001).

**Figure 2:**
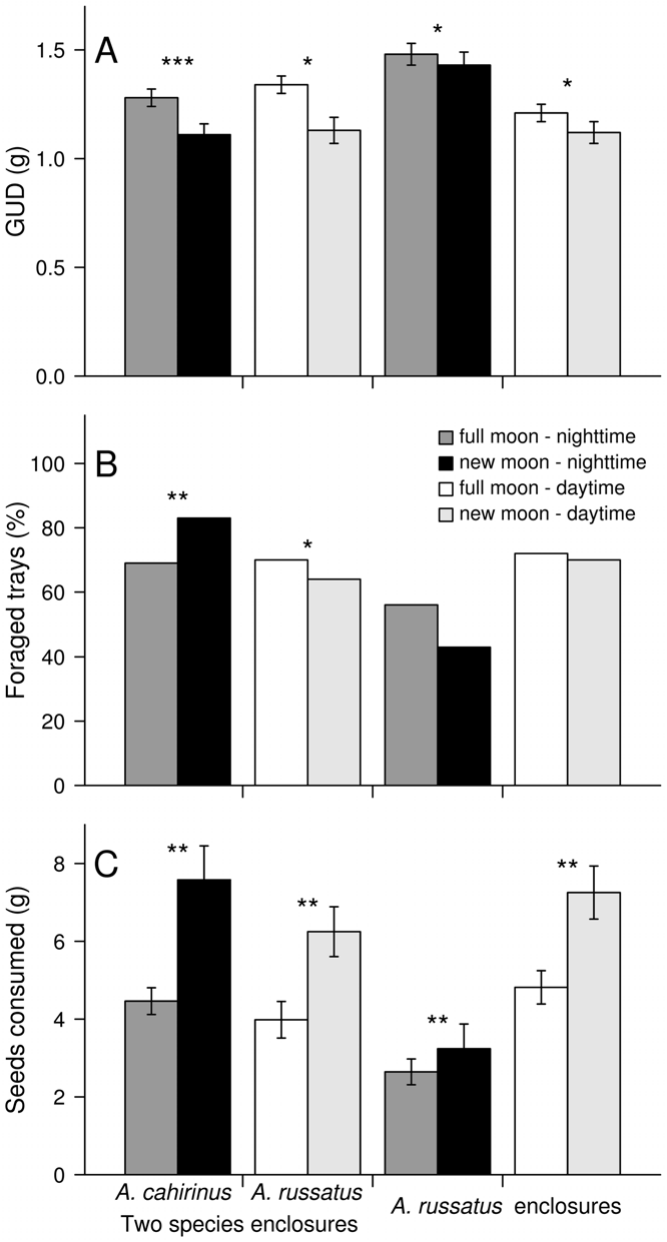
Mean GUDs (g ± SE, A) Percent of trays foraged (B), and total seeds consumption (g ± SE, C) of *A. russatus* and *A. cahirinus* in the two species enclosures and *A. russatus* enclosures during full moon nights and the following days. In the two species enclosures nocturnal foraging is ascribed to *A. cahirinus* and diurnal foraging to *A. russatus*. In the *A. russatus* enclosures both diurnal and nocturnal foraging were carried out by *A. russatus* * p<0.05, ** p<0.01, ***p<0.001. A: Moon phase influenced GUDs, even during daytime: patches were foraged by *A. russatus* to significantly lower GUDs under new moon vs. full moon during both parts of the diel cycle. However, patches were foraged to a significantly lower GUDs during daytime than during nighttime. *A. cahirinus* also foraged to significantly lower GUDs under new moon. The effect of moon was significantly higher on *A. cahirinus* compared to *A. russatus*. B: Moon phase had a significant effect on patch foraging probabilities only in the two species enclosures, where during daytime *A. russatus* patch foraging probabilities were lower during new moon than during full moon. During nighttime *A. cahirinus* patch foraging probabilities were higher during new moon than full moon nights. In the *A. russatus* enclosures patch foraging probabilities of *A. russatus* were lower during nighttime compared to daytime, in which *A. russatus* foraged in fewer patches during nighttime regardless of moon phase. C: Moon phase influenced the amount of seeds eaten at each enclosure: *A. russatus* consumed a higher amount of seeds under new moon vs. full moon during both parts of the dial cycle. However, significantly less amount of seeds were eaten during *A. russatus* nocturnal foraging than during diurnal foraging. *A. cahirinus* also consumed a higher amount of seeds under new moon. Moreover, the effect of moon was not significantly different between the species.

Moon phase influenced patch foraging probabilities at the two species enclosures and influenced foraging efficiency in both treatments and in both diel parts (Figure 2, Table 1). Based on the results of Gutman et al [42], nocturnal foraging in the two species enclosures was attributed to *A. cahirinus* while diurnal foraging in the two species enclosures and all foraging in the *A. russatus* enclosures is attributed to *A. russatus*. Therefore, it can be concluded that moon phase influenced patch foraging probabilities and foraging efficiency of both *Acomys* species (Figure 2). Specifically, moon phase had a significant effect on patch foraging probabilities only in the two species enclosures, where during daytime *A. russatus* patch foraging probabilities were lower during new moon than during full moon (Moon (new) main effect: 0.59±0.62; Moon (new) * Competition (yes): −2.13±0.90; Moon (new) effect in the presence of interspecific competition: −1.56±0.64), while during nighttime *A. cahirinus* patch foraging probabilities were higher during new moon than full moon nights (Moon (new) effect: 0.87±0.29). Hence, in the two species enclosures, moon phase effect was significantly different between the species (Δ_new moon effect [*A. cahirinus* – *A. russatus*]_ = −2.43±0.71). Patch foraging probabilities of *A. russatus* were lower during nighttime compared to daytime (day part [night] effect: −3.13±0.65) in which *A. russatus* foraged in fewer patches during nighttime regardless of moon phase (Figure 2, Table 1).

**Table 1:** Results of the mixed-effects generalized linear mixed-effects models (GLMM) (binomial data) for presence and absence of foraging on trays, the linear mixed-effects models (LMM) for the GUD, and LMM for the amount of seeds eaten of *A. russatus* and *A. cahirinus* in the two species enclosures and *A. russatus* enclosures during full moon nights and the following days.

Test		Estimate	SD	95% CI	99% CI	99.5% CI	Significance
Foraged	*A. cahirinus*						
trays (log odds)	Intercept	0.30	0.60	−0.44, 2.05	−1.42, 3.90	−2.97, 4.69	NS
	New moon effect	0.87	0.29	0.31, 1.46	0.12, 1.64	−0.09, 1.84	**
	*A. russatus*						
	Intercept	0.21	1.86	−4.96, 3.88	−7.16, 6.32	−9.31, 8.65	NS
	New moon	0.59	0.62	−0.64, 1.79	−1.00, 2.20	−1.39, 2.60	NS
	Night	−3.13	0.65	−4.45, −1.89	−4.91, −1.58	−5.39, −1.07	***
	Competition	−0.01	3.20	−6.66, 5.55	−16.69, 11.02	−25.27, 18.05	NS
	moon(new) and daypart(night)	−1.05	0.76	−2.54, 0.45	−3.05, 0.89	−3.53, 1.36	NS
	moon(new) and competition(yes)	−2.13	0.90	−3.97, −0.45	−4.57, 0.07	−5.51, 0.73	*
	Δ_new moon effect [*A. cahirinus* – *A. russatus*]_	−2.43	0.71	−4.96, 3.88	−7.16, 6.32	−9.31, 8.65	NS
Giving Up	*A. cahirinus*						
Densities	Intercept	0.29	0.18	−0.11, 0.62	−0.40, 1.00	−0.71, 1.27	NS
(GUD, g)	New moon effect	−0.30	0.05	−0.4, −0.21	−0.43, −0.18	−0.46, −0.13	***
	*A. russatus*						
	Intercept	0.29	0.19	−0.12, 0.70	−0.49, 1.02	−0.80, 1.40	NS
	New moon	−0.13	0.05	−0.23, −0.03	−0.26, 0.0004	−0.33, 0.03	*
	Night	0.39	0.05	0.29, 0.49	0.26, 0.52	0.23, 0.56	***
	Competition	0.12	0.30	−0.44, 0.69	−1.04, 1.30	−2.34, 1.90	NS
	moon(new) and daypart(night)	0.05	0.08	−0.10, 0.21	−0.15, 0.27	−0.22, 0.33	NS
	moon(new) and competition(yes)	−0.06	0.07	−0.20, 0.08	−0.25, 0.12	−0.30, 0.18	NS
	Δ_new moon effect [A. cahirinus – A. russatus]_	−0.17	0.07	−0.30, −0.03	−0.35, 0.01	−0.41, 0.08	*
Consumed	*A. cahirinus*						
Seeds (g)	Intercept	1.49	1.18	−0.35, 3.56	−3.97, 6.53	−12.05, 10.82	NS
	New moon effect	3.00	1.00	1.07, 4.92	0.30, 5.65	−0.81, 7.20	**
	*A. russatus*						
	Intercept	1.56	1.14	−0.23, 3.21	−4.21, 5.47	−12.56, 11.94	NS
	New moon	2.37	0.72	0.95, 3.77	0.45, 4.22	−0.65, 5.22	**
	Night	−2.55	0.46	−3.44, −1.64	−3.76, −1.31	−4.12, −0.83	***
	Competition	−0.83	2.06	−4.76, 3.02	−8.57, 7.77	−16.59, 16.51	NS
	moon(new) and daypart(night)	−1.73	1.05	−3.77, 0.35	−4.44, 1.10	−5.50, 2.60	NS
	moon(new) and competition(yes)	−0.18	1.02	−2.15, 1.75	−2.83, 2.47	−3.82, 3.94	NS
	Δ_new moon effect [A. cahirinus – A. russatus]_	0.63	1.23	−1.74, 3.02	−2.58, 3.79	−3.82, 5.61	NS

In the two species enclosures nocturnal foraging is ascribed to *A. cahirinus* and diurnal foraging to *A. russatus*. In the *A. russatus* enclosures both diurnal and nocturnal foraging were carried out by *A. russatus*. NS: 95% CI span zero.

*95% CI don't span zero,

**99% CI don't span zero,

***99.5% CI don't span zero.

Moon phase also influenced GUDs, even during daytime (Figure 2, Table 1): patches were foraged by *A. russatus* to significantly lower GUDs under new moon vs. full moon (Moon (new) effect:−0.13±0.05) during both parts of the dial cycle (no significant interaction with day part term, 0.05±0.08). No significant main effect of competition was found (Competition (yes) effect: 0.12±0.30). However, patches were foraged to a significantly lower GUDs during daytime than during nighttime (day part (night) effect: 0.39±0.05). No significant day-part * moon phase interaction (0.05±0.08) was found. *A. cahirinus* also foraged to significantly lower GUDs under new moon (Moon (new) effect: −0.30±0.05). Moreover, the effect of moon was significantly higher on *A. cahirinus* compared to *A. russatus* (Δ_new moon effect [*A. cahirinus* – *A. russatus*]_ = −0.17±0.07).

Moon phase also influenced the total amount of seeds eaten at each enclosure at both daytime and nighttime (Figure 2, Table 1): significantly more seeds were consumed by *A. russatus* under new moon vs. full moon (Moon (new) effect: 2.37±0.72) during both parts of the dial cycle (no significant interaction with day part term, −1.73±1.05). Competition had no significant main effect (Competition (yes) effect: −0.83±2.06). However, *A. russatus* consumed significantly more seeds during daytime than during nighttime (day part (night) effect: −2.55±0.46). There was no significant day-part * moon phase interaction (−1.73±1.05). *A. cahirinus* also consumed significantly more seeds under new moon (Moon (new) effect: 3.00±1.00). The effect of moon was not significantly different between *A. cahirinus* and *A. russatus* (Δ_new moon effect [*A. cahirinus* – *A. russatus*]_ = 0.63±1.23).

## Discussion

Moon phase affected fecal cortisol metabolite levels and foraging behavior of populations of both species in all experimental conditions; fecal cortisol metabolite levels were elevated as were GUDs.

During full moon nights, *A. cahirinus* visited fewer trays than during new moon nights; the opposite pattern occurred in *A. russatus*, but it was significant only in the two species enclosures (where the species is diurnal). Nevertheless, the total amount of seeds consumed was lower in full moon nights and the days following them for all populations, indicating a significant effect of moon phase on foraging behavior. The fact that in spite of their foraging in more patches, total seeds consumed was lower during full moon nights suggests two possible scenarios: a) that *A. russatus* had shorter visits to each tray, resulting in shorter foraging time per patch and therefore higher GUD values; b) similar time was spent on each patch, but more time was devoted to vigilance (in response to the elevated perceived predation risk [81]), resulting in less efficient foraging in each patch. Be that as it may, the increase in the number of trays foraged during full moon night or days did not compensate for the increased GUDs.

The behavioral response may be mediated, at least in part, by the elevated cortisol levels, reflected in our study by change in fecal cortisol metabolite levels, as has previously been demonstrated under laboratory settings. GCs were shown to have an effect on different behaviors that may result in reduced foraging. For example, in a light dark box test, GC treatment resulted in increased latency in the dark compartment, and in the elevated plus maze (elevated maze with two exposed and two sheltered arms) it increased time spent in the sheltered arms [31]–[34]. Tree lizards treated with GC responded more quickly to the predator and hid longer than control lizards [35], and in the Adelie penguin (*Pygoscelis adeliae*) individuals with high pre-foraging corticosterone levels spent less time foraging, and stayed closed to the colony than penguins with low pre-foraging corticosterone levels [82], so baseline corticosterone levels were correlated with foraging behavior. We suggest that elevated GC levels in spiny mice resulted in increased anxiety and fear, which influenced their foraging behavior. As mentioned earlier, GC may also affect foraging behavior via their effect on food consumption, so one could speculate that it is GC levels directly reducing consumption. However, since the relationships between GC levels and food consumption are complex, this cannot be determined from this study.

*A. russatus* are active in nature only during the day and have probably been so for millennia [42], [66], so individuals in the wild and in our two species enclosures do not routinely experience predation risk by owls, nor have their ancestors. The fact that when *A. russatus* revert to nocturnal activity, they respond to moon phase both in hormone levels and foraging behavior, suggests that this ‘hard-wired’ response is evolutionarily constrained; *A. russatus* individuals respond to elevated illumination although in all likelihood they have never faced risk of owl predation, suggesting that this response is genetically determined and was not lost in spite of the shift in activity patterns [46], [63], [67], [68]. Previous research shows that although *A. russatus* are diurnally active and have even evolved some adaptations for this activity pattern (reviewed by [42], [66]), they retain various physiological and morphological traits of nocturnal mammals [46], [61], [63], [64], [68], [69].

Behavior is considered to be of great evolutionary plasticity – changing prior to genetic changes; “in circumstances where rapid reaction to environmental events is required, behavior must invariably be more flexible than genetic change” [83]. *A. russatus'*s behavioral rigidity indicates that avoidance behavior, reduced foraging during moonlit nights, is extremely stable to the point that it is retained in a diurnally active rodent.

More surprising is the fact the diurnal populations of *A. russatus* also respond to moon phase. Since predation efficiency of diurnal predators is not expected to be influenced by moon phase the previous night, moon phase is not expected to affect predation risk during the subsequent day, so diurnal moon-phase related behavior is probably not adaptive. In fact, it is the opposite of that expected if diurnal foraging should compensate for reduced nocturnal foraging, as has been described in several studies of other species: increased activity at dusk or dawn during full moon nights (*Dipodomys merriami*, [16]), more evenly spread activity in response to a predator in enclosed bank voles (*Clethrionomys glareolus*, [84]), increased diurnal activity bouts after exposure to light intensities similar to full moon light (*Phyllotis xanthopygus*, [85]), and inversion in activity patterns of Norwegian rats at high red fox densities [86]. How can we explain the response of diurnal *A. russatus* to moon phase?

Root voles (*Microtus oeconomus*) are active during both day and night; Halle [87] found that they reduced their activity in response to full moon nights and also during the days following them, “suggesting possible lunar periodicity in behavior of root voles” [87]. However, experiments carried out in controlled conditions demonstrate that rodents, including *A. cahirinus*, respond behaviorally to elevated illumination regardless of natural moon-phase [8], [13], [26], [88], [89]. If this response is mediated by GC, then GC should have lunar periodicity, but such endogenous periodicity was never described in mammals; lunar periodicity is currently known only in marine species, apparently a response to physical changes in tides that result from moon phase shifts (reviewed by [90]).

An alternative hypothesis can be based on the concept of a “memory window” [91] in which an optimal forager's knowledge of the environment and thereby its behavior are affected by the recent past; i.e., by a running average of the prior×patches (or t-times) [92]. Thereafter patch profitability is compared against this updated parameter rather than the unknown global value [92]. Perhaps *A. russatus* exhibited reduced foraging efficiency during days that followed moonlit nights because of a “memory” of increased risk of predation during the full moon night. This hypothesis is in accord with the preparative action of GCs [27]. Working with laboratory rats and mouse strains, which never encountered a predator, investigators use stimuli such as predator odor and smell, and even light to induce stress response. These studies rely on existence of an association between the stimulus, and the actual stressor. Such association is widely used in the study of stress: exposure to pairs of emotionally neutral stimuli such as sound, coupled with aversive stimuli, triggers a learning process, resulting in an acquired stress response to the neutral stimuli [93]. Such associations exist in animals that were never exposed to the actual stressor (e.g., individuals that were never exposed to a predator respond to its odor), suggesting that they are genetically determined, and can explain the mechanism for the “memory window” resulting in increased GC levels and reduced foraging in diurnally active *A. russatus*.

Indeed experiments of predator-prey interactions have shown the significance of previous experience [28]. For example, mice exposed to a live cat remained in their burrows for over 14 h [94], and an impaired long-term memory of 16–22 days followed the predatory stress [95]. Bank voles reduced activity when enclosed with a weasel, and recovered only days after its removal [84]. Kotler [56] found that gerbils had a slow rate of recovery (1–5 days) in foraging activity after being held with a barn owl. Sih et al. [29] point that prey may have difficulty in detecting and responding to a decrease in risk.

In sum, we found that both species exhibit high fecal cortisol metabolite levels and reduced foraging when the moon is full. We suggest that reduced foraging may be mediated by increased GC levels. More surprising are increased GC levels and reduced foraging in diurnally active *A. russatus* during days that follow moonlit nights. Not only does this behavior have no straightforward adaptive value, it can reduce fitness of these individuals (resulting from decreased food consumption and higher GC levels). We suggest that GC levels increase in response to previous experience – elevated risk of predation during moonlit nights, and that these elevated hormone levels induce predator-avoidance behavior in the field. Hormonal and behavioral responses to predation risk appear ‘hard wired’ in *A. russatus*, remaining evolutionarily constrained in spite of the shift in activity patterns.

## Supporting Information

Figure S1 The effect of trapping on mean fecal cortisol metabolite levels (µg/dL ± SE) in (A) *A. russatus* and (B) *A. cahirinus* (experimental group – filled bars, n = 15, control group – empty bars, n = 15). * - P<0.05.
(TIFF)

Figure S2 Daily rhythms in mean fecal cortisol metabolites level (○, % ± SE), relative activity levels (×, % ± SE) and body temperature (•, °C ± SE) of *A. russatus* (A, n = 10) and *A. cahirinus* (B, n = 9). Data for fecal cortisol metabolite levels and activity levels are presented as % of the highest value obtained for each individual. Dark background represents the dark hours.
(TIFF)

Figure S3 The relationship between increasing pooled fecal mass (g) of *A. russatus* (A) and *A. cahirinus* (B) extracted and fecal cortisol metabolite levels (µg/dL). Dashed line represents the regression line: *A. russatus*– R^2^ = 0.83, p<0.01; *A. cahirinus* – R^2^ = 0.86, p<0.01).
(TIFF)

Text S1 Validation of fecal cortisol measurements.
(DOC)
