# Supplementary figures and images for: The Effect of the Lunar Cycle on Fecal Cortisol Metabolite Levels and Foraging Ecology of Nocturnally and Diurnally Active Spiny Mice
Source: PLoS One. 2011 Aug 4;6(8):e23446. doi: 10.1371/journal.pone.0023446 (PMC3150436; doi:10.1371/journal.pone.0023446)

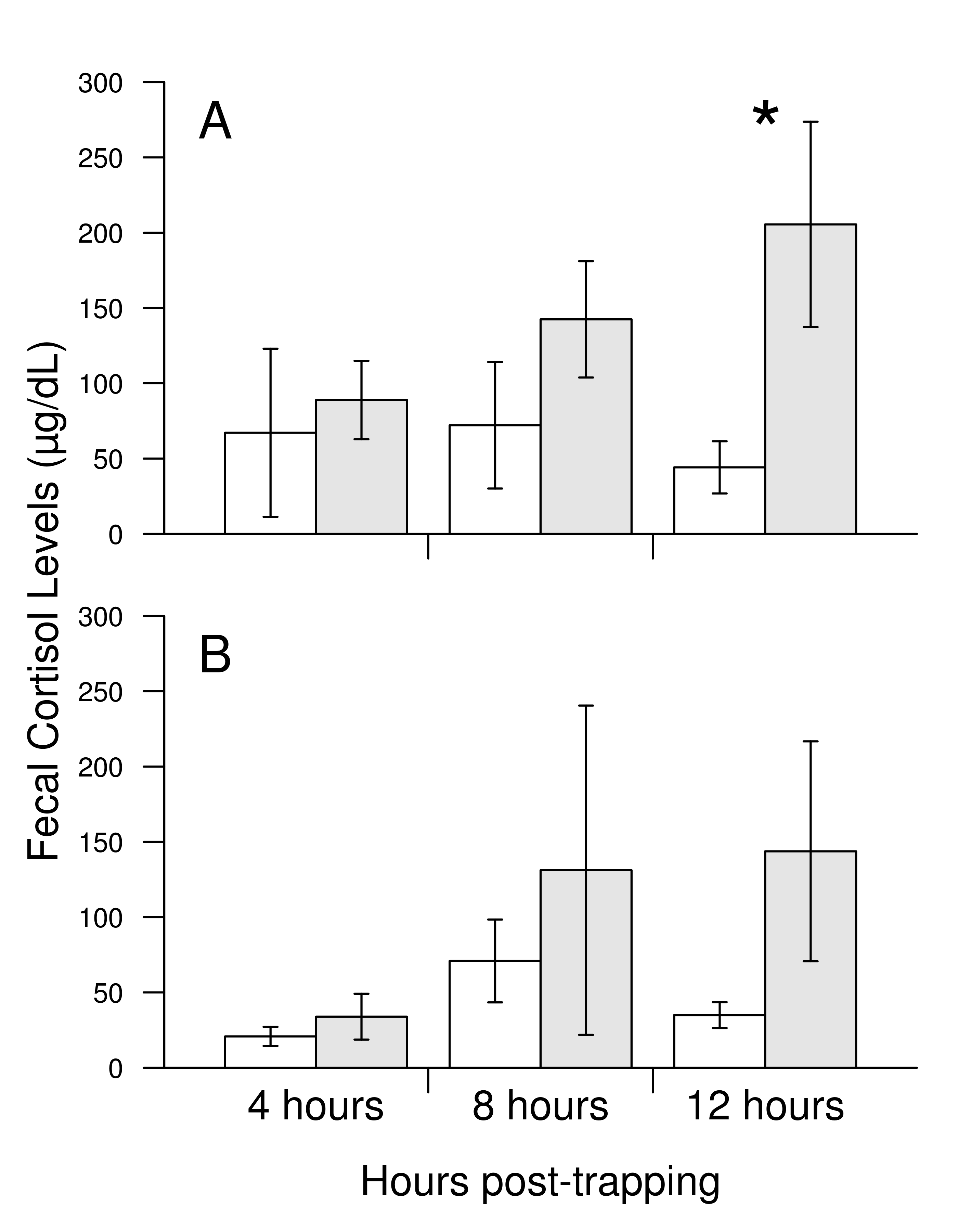

Supplement: Figure S1 — The effect of trapping on mean fecal cortisol metabolite levels (µg/dL ± SE) in (A) A. russatus and (B) A. cahirinus (experimental group – filled bars, n = 15, control group – empty bars, n = 15). * - P<0.05. (TIFF) [file pone.0023446.s001.tiff]

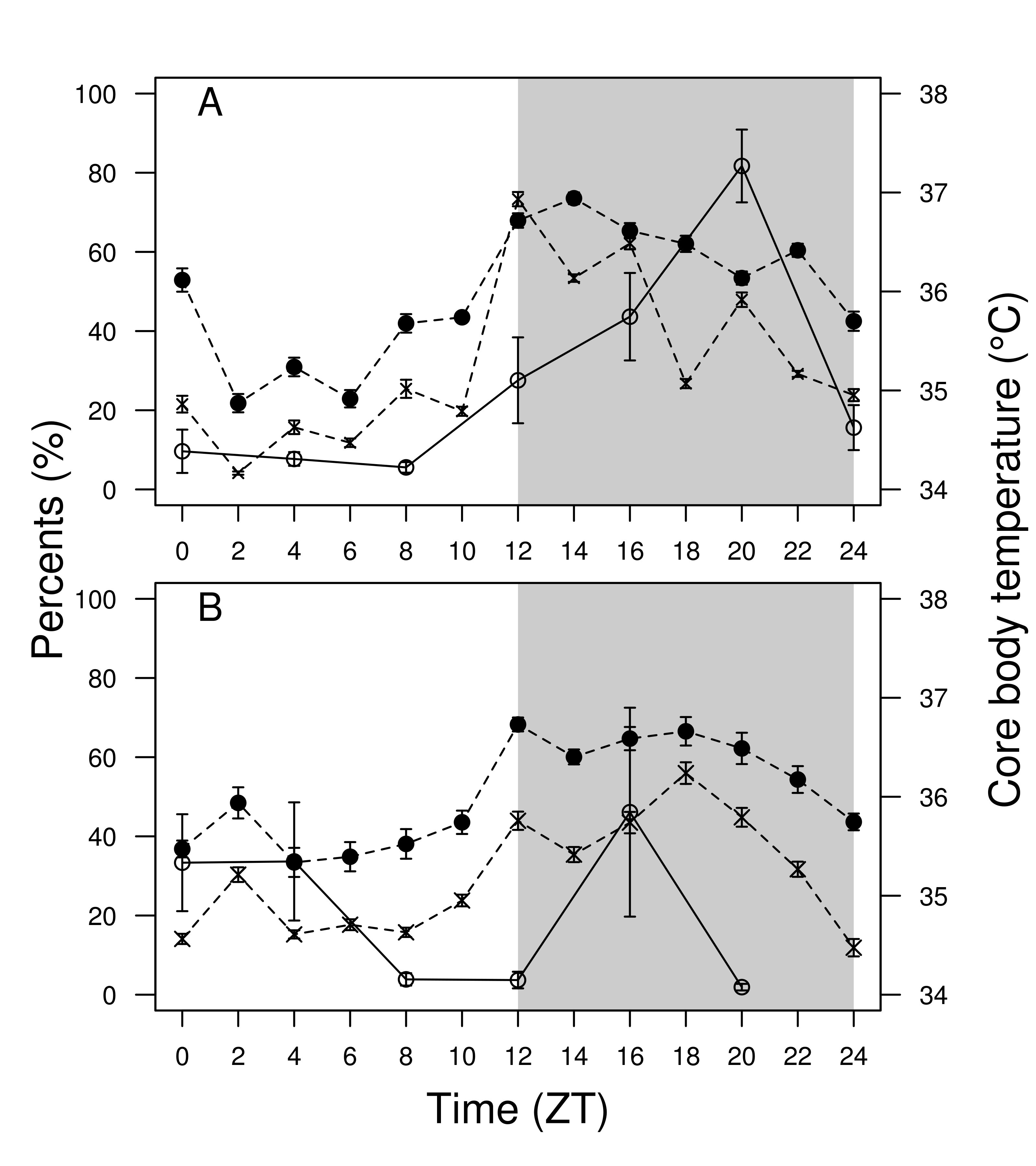

Supplement: Figure S2 — Daily rhythms in mean fecal cortisol metabolites level (○, % ± SE), relative activity levels (×, % ± SE) and body temperature (•, °C ± SE) of A. russatus (A, n = 10) and A. cahirinus (B, n = 9). Data for fecal cortisol metabolite levels and activity levels are presented as % of the highest value obtained for each individual. Dark background represents the dark hours. (TIFF) [file pone.0023446.s002.tiff]

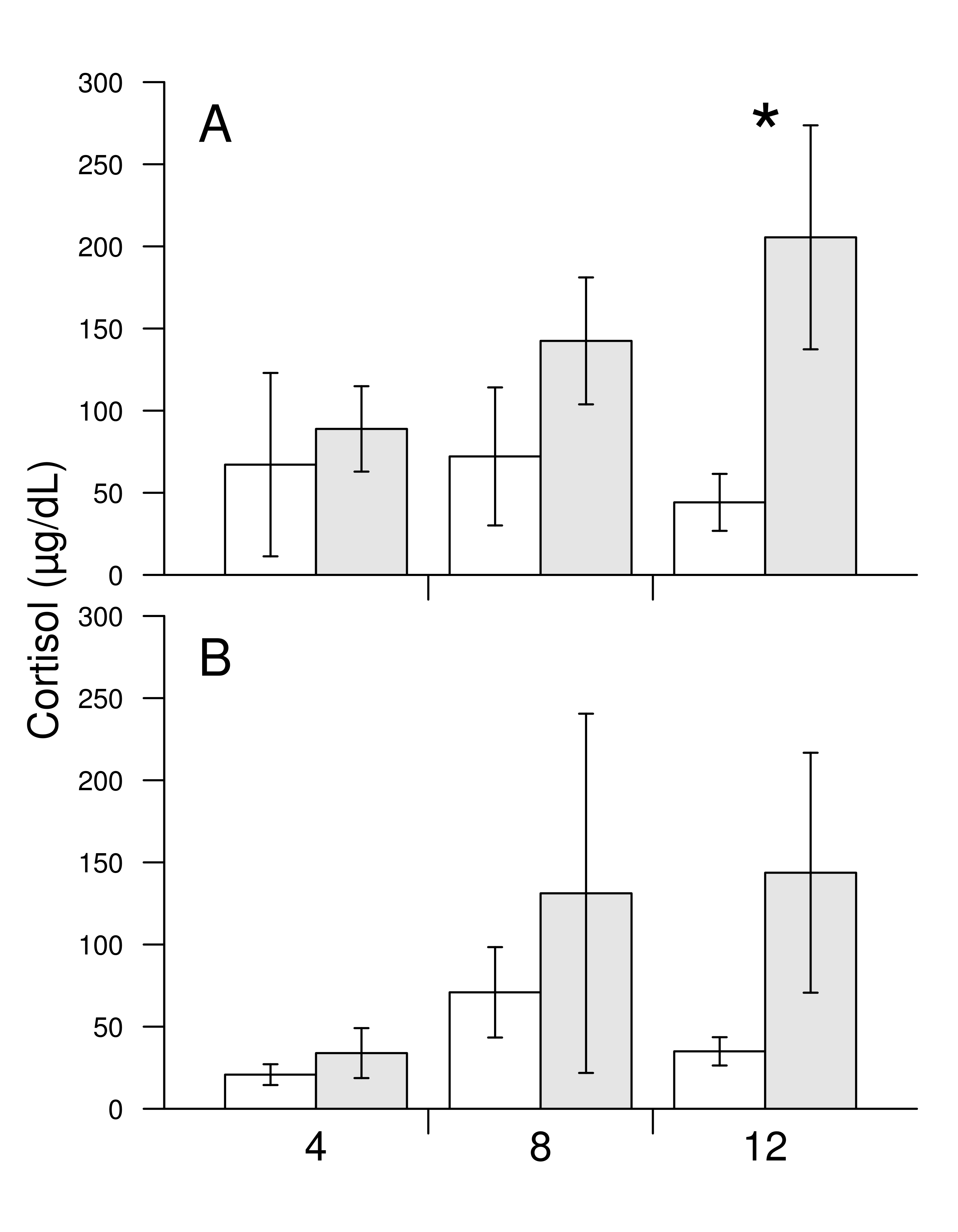

Supplement: Figure S3 — The relationship between increasing pooled fecal mass (g) of A. russatus (A) and A. cahirinus (B) extracted and fecal cortisol metabolite levels (µg/dL). Dashed line represents the regression line: A. russatus– R2 = 0.83, p<0.01; A. cahirinus – R2 = 0.86, p<0.01). (TIFF) [file pone.0023446.s003.tiff]
