# Supplementary material for: The Effect of the Lunar Cycle on Fecal Cortisol Metabolite Levels and Foraging Ecology of Nocturnally and Diurnally Active Spiny Mice
Source: PLoS One. 2011 Aug 4;6(8):e23446. doi: 10.1371/journal.pone.0023446 (PMC3150436; doi:10.1371/journal.pone.0023446)
Supplement: Text S1 — Validation of fecal cortisol measurements. (DOC) [file pone.0023446.s004.doc]

**Validation of fecal cortisol measurements**:

**METHODS**

Experiments were conducted using animals from our breeding colonies of *A. russatus* and *A. cahirinus*, originally trapped near the Dead Sea, Israel, and kept at the Meier I. Segals Garden for Zoological Research at Tel Aviv University (permit number 2003 / 16295). All procedures were conducted in accordance with the Institutional Animal Ethics Committee's regulation.

**1. Serial sampling before and after a stressful event***:*  30 individuals of each species (both males and females, ratio 1:1) were used in this study. Each individual was housed in a standard plastic cage (21x31x13 cm), in a controlled room under a 12:12 light:dark cycle. Room temperature was 30 ± 1°C for *A. russatus* and 28 ± 1°C for *A. cahirinus* (approximately the low critical temperature of the thermoneutral zone of each species [1]). Water and standard rodent pellets (Koffolk serial no. 19510: protein - 21%, fat - 4%, carbohydrates - 75%, RQ= 0.96) were provided *ad libitum*. After a three week acclimation period, animals were divided into three groups (n=10 in each group). Each group was further divided into control and experimental group (n=5 in each), so in total we had 3 experimental groups and three control groups. On the first day, each individual of the experimental groups was trapped in a clean Sherman trap for 4, 8 or 12 hours, while control individuals were transferred to a clean cage for the same period of time. We repeated the experiment three times, three days apart, alternating the time spent in the traps (or cages for the control groups) between the groups, so at the end of the experiment each experimental or control individual stayed in the trap (or cage) for all three periods of time (in total we had 15 individuals in each control or experimental group at each time point). At the end of each trapping session, all feces from the trap or cage were collected, and stored in 95% ethanol in –20^o^C until extraction and assay as described in the MS.

.

**2. Describing the naturally occurring diurnal variation in** **fecal glucocorticoid metabolites:**

Ten *A. russatus* and 9 *A. cahirinus* were used in this study. Each individual was implanted with either DSI biotelemetry transmitters or Mini Mitter E-Mitters in the abdominal cavity (see below), and housed in a standard plastic cage (21x31x13 cm), in a controlled room under a 12:12 light:dark cycle. Room temperature was 30 ± 1°C for *A. russatus* and 28 ± 1°C for *A. cahirinus*. Water and standard rodent pellets were provided *ad libitum.* After a three week recovery and acclimation period, feces were collected every 4 hours, for 24 hours. Each mouse had 2 cages, and was transferred between them every 4 hours. After moving an individual to its alternate cage, all feces from the cage were collected and stored in 95% ethanol in –20°C until extraction and assay as described in the MS.

Body temperature and activity were continuously recorded using either an integrated telemetry and data acquisition system (Dataquest III, Data Sciences Inc., St. Paul, MN, USA) with gross activity and temperature-sensing transmitter (TA10TA-F20, Data Sciences, Inc., St. Paul, MN, USA) for *A. russatus*, or a VitalView Data Acquisition System (Respironics Mini Mitter, OR, USA) with a gross activity and temperature sensing E-Mitter (G2 E-Mitter, Respironics Mini Mitter, OR, USA) for *A. cahirinus*.

Animal surgery: Mice were anesthetized with isoflorane in medical grade oxygen using an anesthetic machine (Ohmeda, 1.5% vol, 1 L/min) and implanted with either DSI biotelemetry transmitters or Mini Mitter E-Mitters in the abdominal cavity. Both the abdominal wall and the skin were sutured with absorbable surgical suture, with cutting needle (5-0 Dexon) and the incision was treated with topical antibiotic (silver sulfadiazine 1%; Silverol Cream). Prophylactic antibiotics (Baytril 5% 24 mg/kg) and artificial tear ointment (to prevent desiccation) were administered preoperatively. Body temperature and activity data were collected in 6 min. intervals, and averaged per hour. We tested the significance of the 24 h variation in fecal cortisol metabolite levels using one way mixed effects ANOVA with individuals as the random factor.

**3. Parallel dilution**: a stock of concentrated feces extract was diluted in increased amount of assay buffer, and assayed as described in the MS.

**4. Extracting increasing amounts of feces:** we extracted an increasing amount of homogenized fecal matter, and assayed as described in the MS.

**RESULTS:**

**1. Serial sampling before and after a stressful event***:*  trapping stress increased fecal cortisol metabolite levels after 8-12 h (Figure S1). In *A. russatus*, fecal stress hormone levels were significantly higher in the experimental group after 12 hours (one tail paired t-tests: 4 hr: t=-0.33, df=14, p=0.37; 8 hr: t=-1.15, df=14, p=0.13; 12 hr: t=-2.61, df=11, p<0.05). In *A. cahirinus*, the difference between control and experiment of fecal stress hormone levels was the highest after 12 hr, although is was only marginally significant (one tail paired t-tests: 4 hr: t=-0.81, df=14, p=0.21; 8 hr: t=-0.33, df=13, p=0.51; 12 hr: t=-1.49, df=13, p=0.07).

**2. Diurnal variation in the fecal glucocorticoid metabolites**: both species had a significant daily variation in fecal cortisol metabolite levels,(*A. russatus*: df=7, F=25.0, p<0.001; *A. cahirinus*: df=6, F=3.3, p<0.05, Figure S2). As in previous experiments, under laboratory conditions, both species were active, and their body temperature was higher during the dark. The peak in fecal cortisol metabolite levels occurred 8 and 4 hours after dark onset in *A. russatus* and *A. cahirinus* respectively, in accord with the expected peak just before activity onset and the 8-12 hours delay in the appearance of the metabolites in the feces (see experiment 1).

**3. Parallel dilution**: cortisol (from standard solutions) and cortisol-like immuno-reactivity in spiny mice feces extract diluted in parallel in the ICN cortisol RIA (p = 0.0052, R^2^ = 0.817 for *A. russatus* and p = 0.0083, R^2^ = 0.828 for *A. cahirinus*).

**4. Extracting increasing amounts of feces:** fecal mass correlated to cortisol-like immuno-reactivity in both species (simple regression analysis: *A. russatus* mass effect – 1430.9 ± 291.1, R^2^=0.83, t=4.9, df=5, p<0.01; *A. cahirinus* mass effect – 698.3 ± 127.1, R^2^=0.86, t=5.5, df=5, p<0.01; [μg/dL±SD]) (Figure S3).

Reference

1. Shkolnik A, Borut A (1969) Temperature and water relations in 2 species of spiny mice (*Acomys*). J Mammal 50: 245-255.
